# Stimulation of B Cell Immunity in Flavivirus-Naive Individuals by the Tetravalent Live Attenuated Dengue Vaccine TV003

**DOI:** 10.1016/j.xcrm.2020.100155

**Published:** 2020-12-22

**Authors:** Huy A. Tu, Usha K. Nivarthi, Nancy R. Graham, Philip Eisenhauer, Matthew J. Delacruz, Kristen K. Pierce, Stephen S. Whitehead, Jonathan E. Boyson, Jason W. Botten, Beth D. Kirkpatrick, Anna P. Durbin, Aravinda M. deSilva, Sean A. Diehl

**Affiliations:** 1 Department of Microbiology and Molecular Genetics, Vaccine Testing Center, Larner College of Medicine, University of Vermont, Burlington, VT 05405, USA; 2 Cellular, Molecular, and Biomedical Sciences Graduate Program, University of Vermont, Burlington, VT 05405, USA; 3 Department of Microbiology and Immunology, University of North Carolina School of Medicine, Chapel Hill, NC 27599, USA; 4 Department of Medicine, Larner College of Medicine, University of Vermont, Burlington, VT 05405, USA; 5 Laboratory of Viral Diseases, National Institute of Allergy and Infectious Diseases, National Institutes of Health, Bethesda, MD 20892, USA; 6 Department of Surgery, Larner College of Medicine, University of Vermont, Burlington, VT 05405, USA; 7 Department of International Health, Center for Immunization Research, Johns Hopkins Bloomberg School of Public Health, Baltimore, MD 21205, USA

**Keywords:** dengue vaccine, tetravalent live attenuated vaccine, humoral immune response, neutralizing antibodies, memory B cells, CD4 T cells, protective immunity, TV003, serotype-specific antibodies

## Abstract

The tetravalent live attenuated dengue vaccine candidate TV003 induces neutralizing antibodies against all four dengue virus serotypes (DENV1–DENV4) and protects against experimental challenge with DENV2 in humans. Here, we track vaccine viremia and B and T cell responses to this vaccination/challenge model to understand how vaccine viremia links adaptive immunity and development of protective antibody responses. TV003 viremia triggers an acute plasmablast response that, in combination with DENV-specific CD4^+^ T cells, correlates with serum neutralizing antibodies. TV003 vaccinees develop DENV2-reactive memory B cells, including serotype-specific and multivalent specificities in line with the composition of serum antibodies. There is no post-challenge plasmablast response in vaccinees, although stronger and earlier post-TV003 plasmablast responses associate with sterile humoral protection from DENV2 challenge. TV003 vaccine triggers plasmablasts and memory B cells, which, with support from CD4^+^ T cells, functionally link early vaccine viremia and the serum antibody responses.

## Highlights

The tetravalent live attenuated dengue vaccine TV003 stimulates plasmablasts
Robust plasmablast response is associated with sterile protection from challenge
DENV-specific memory B cells persist 6 months after vaccination
DENV-specific CD4^+^ T cells correlate with neutralizing antibodies

## Graphical Abstract

**Figure:**
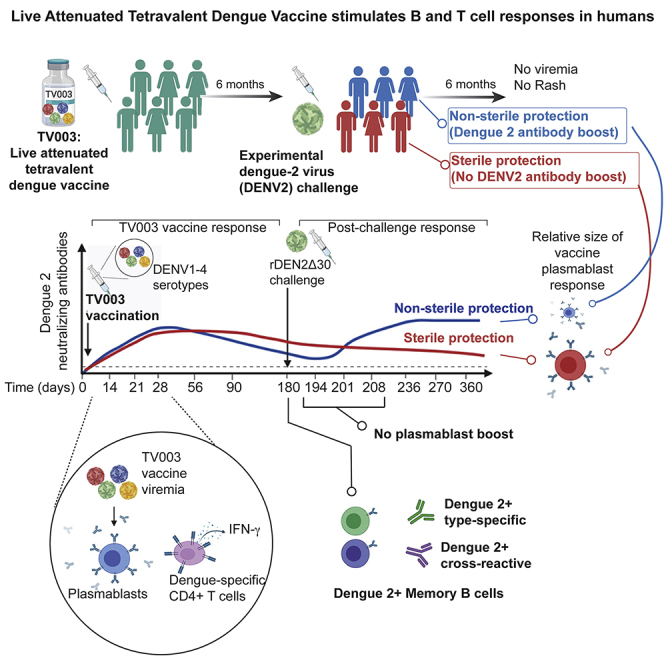

## Teaser

Tu et al. show that the protective live attenuated tetravalent dengue vaccine stimulates early B and T cell responses to promote durable memory and neutralizing antibodies.

## Introduction

The four serotypes of the dengue virus (DENV1–DENV4) cause an estimated 390 million infections every year, with ∼100 million clinically apparent cases.^1^ Dengue disease ranges from non-specific febrile illness with rash and body aches to more severe symptoms, including hemorrhagic fever or shock syndrome.^2^ Infection by one serotype can confer lifelong protection against subsequent symptomatic homotypic infections. After a short window of cross-protection following the primary infection, a heterotypic secondary infection can be associated with severe dengue disease.^3^^,^^4^ Thus, it is critical that a dengue vaccine promotes robust and balanced immunity to all DENV serotypes to provide maximum protection and minimize the risk for secondary infection-associated disease.

The most clinically advanced dengue vaccine candidates are tetravalent live attenuated vaccines.^5^ Each includes the structural genes coding for prM (pre-membrane) and E (envelope) proteins from each of the four DENV serotypes. The E protein, which decorates the surface of the viral particle and mediates particle attachment and entry into host cells, is thought to be the major target of the anti-DENV neutralizing antibody response. Serum neutralizing antibodies have been the primary metric for the evaluation of dengue vaccine immunogenicity.^6^ Serum neutralizing antibodies correlate with protection for other flavivirus vaccines against yellow fever and Japanese encephalitis viruses,^7^ but may be inadequate to explain protection for all dengue vaccines. The chimeric yellow fever/dengue (CYD) vaccine (i.e., yellow fever virus backbone with the prM and E proteins of DENV) induced a high rate of seropositivity (as assessed by serum neutralizing antibodies) to multiple serotypes, but protection varied widely across serotypes and occurred mainly in subjects that were dengue seropositive at the time of vaccination.^8^, ^9^, ^10^, ^11^ Similarly, baseline serostatus appears to influence serum neutralizing antibody levels to other tetravalent live attenuated dengue vaccines.^12^^,^^13^

The tetravalent live attenuated dengue vaccine candidate TV003 from the National Institute of Allergy and Infectious Diseases has progressed though Phase I and II clinical studies, including in endemic areas, where it has proven to be safe and able to induce neutralizing antibodies against all four DENV serotypes.^14^, ^15^, ^16^, ^17^, ^18^ TV003 is now in a Phase III clinical trial (NCT02406729) in endemic areas. TV003 completely protected flavivirus-naive adult subjects from clinical symptoms and dengue viremia, as measured by virus culture and RT-PCR, following challenge with a recombinant heterotypic DENV2, known as rDEN2Δ30.^19^ Nearly half of the vaccinated cohort exhibited an increase in DENV2 serum neutralizing antibodies after challenge, indicating sterilizing immunity in some subjects. The major goal of our study was to evaluate the kinetics and phenotypic profile of plasmablasts and memory B cells in the setting of protective human DENV vaccination.

Viral antigen exposure during infection or vaccination induces a population of highly proliferative antibody-secreting B cells known as plasmablasts.^20^, ^21^, ^22^, ^23^, ^24^ In our human rDEN2Δ30 primary infection model, we showed that peripheral blood plasmablasts peaked at 2 weeks post-infection, with a preponderance of these cells producing antibodies specific to DENV2.^25^ Furthermore, this DENV2 specificity was maintained in the memory B cell compartment at 6 months post-infection.^25^ We sought to understand how B and T cells are induced by TV003 and how these cells functionally link other vaccine-associated parameters such as vaccine viremia and elicitation of serum antibodies. Here, we used longitudinal serum and peripheral blood samples collected from subjects immunized with TV003 and subsequently challenged with rDEN2Δ30 to ask how key features of the B cell response may correlate with vaccine immunogenicity and protection from challenge. We show TV003 vaccine viremia corresponded with an increase in plasmablasts, which were associated with the development of DENV serum neutralizing antibodies and a broad, durable DENV-specific memory B cell response.

## Results

### Kinetics of Plasmablast Formation following TV003 Vaccination

**Figure 1:**
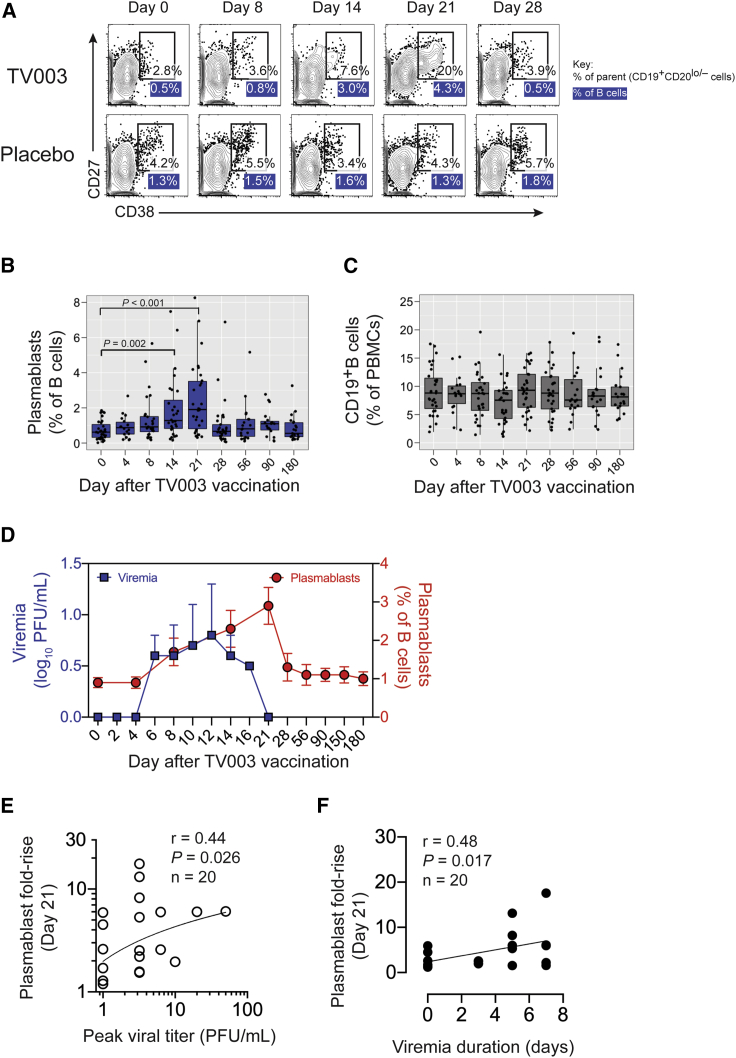
Induction of Plasmablasts by a Live Attenuated Tetravalent DENV Vaccine (A) Plasmablasts were defined at the indicated times after vaccination by flow cytometry as CD38^hi^CD27^hi^ cells within the CD19^+^CD20^low/−^ B cell population in live CD3^−^CD14^−^ PBMCs from TV003 vaccinees. A representative vaccine and placebo sample are shown. Values in the gate refer to the percentage of the CD19^+^CD20^low/−^ parent population, and the blue-shaded values under the gate indicate the percentage of the plasmablasts of total CD19^+^ B cells. (B and C) Frequencies of plasmablasts (B) as a percentage of (C) total CD19^+^ B cells after vaccination with TV003 (n = 30 subjects). Boxplot areas show 25^th^-75^th^ percentiles with the median as a thick line and whiskers indicating the 95% confidence interval and closed circles indicate each data point from each subject. (D) Mean plasmablast frequencies ± standard deviations (SDs) (red circles) and mean vaccine-associated viral titers ± SDs (blue squares) are plotted as a function of day after vaccination with TV003 (n = 20 subjects). (E and F) Correlation of day 21 plasmablast fold-rise (defined as plasmablast frequency at day 21 divided by the frequency at day 0) with (E) duration of viremia and (F) peak viral titer. Spearman R correlation coefficients and p values are reported. At least 2 technical replicates were performed for all of the viremia measurements.

Acute DENV infection has been shown to induce a population of highly proliferative antibody-secreting B cells known as plasmablasts, up to 70% of which are virus specific.^22^^,^^26^, ^27^, ^28^ We previously showed that the increased level of plasmablasts in primary DENV2 infection correlated with viral load and neutralizing antibodies.^25^ We therefore hypothesized that the kinetics and size of the plasmablast pool would be related to vaccine viremia and serum antibodies in the context of vaccination with live attenuated dengue vaccines. To investigate this, we assessed CD38^hi^CD27^hi^ plasmablasts in peripheral blood mononuclear cells (PBMCs) of healthy flavivirus-naive adults following immunization with the tetravalent live attenuated dengue vaccine TV003 (Figure 1A). TV003 induced peak plasmablast frequencies ranging from 0.4% to 8.3% of B cells (median of ∼2%) and occurring at days 4, 8, 14, 21, or 28 after immunization (Figure S1A). Cumulatively, the data showed that plasmablasts were significantly induced on days 14 and 21 after immunization (Figure 1B). Our samples were from 2 different Phase I trials of TV003 from 2010 and 2013–2014, but plasmablast kinetics or magnitude did not differ between the trials (Figure S2A). The increase in plasmablast frequency after TV003 immunization was not due to changes in total CD19^+^ B cells, as these remained stable after vaccination (Figures 1C and S1B).

### Relation of Plasmablasts and TV003 Vaccine Virus Replication

We then focused on plasmablasts in the context of a TV003 vaccination and heterotypic rDEN2Δ30 challenge in samples from the CIR287 trial to ask whether plasmablast dynamics reflected vaccine viral replication or protection from challenge. TV003 vaccine viremia across any serotype was detected by day 4 post-immunization and persisted up to 1 week and waned before the peak plasmablast response was detected (Figure 1D). Point-in-time plasmablast frequencies at days 14 or 21 after vaccination did not correlate with vaccine peak viremia (Figures S3A and S3B) or vaccine viremia duration (Figures S3D and S3E). To account for subject-level variation in baseline and post-vaccination responses (Figure S1A), we calculated the ratio (i.e., fold-rise) of peak to baseline plasmablast frequencies. Given that day 21 after vaccination was the prevailing peak plasmablast response time point (in 17 of 30 vaccinees; Table S1) we calculated fold-rise at day 21 for each subject. Day 21 plasmablast induction positively correlated with peak viral titer (Figure 1E) and with viremia duration (Figure 1F). We also found positive correlations between peak plasmablast induction occurring at any point within 28 days after vaccination (Table S1), with peak vaccine viral titer (Figure S3C), and viremia duration (Figure S3F), but did not find positive correlations of vaccine viremia with day 14 or 21 plasmablast frequencies (Figures S3A, S3B, S3D, and S3E). Overall, these data showed that plasmablast induction positively correlated with TV003 vaccine viral replication.

### TV003-Induced Plasmablasts and Serum-Neutralizing Antibodies

**Figure 2:**
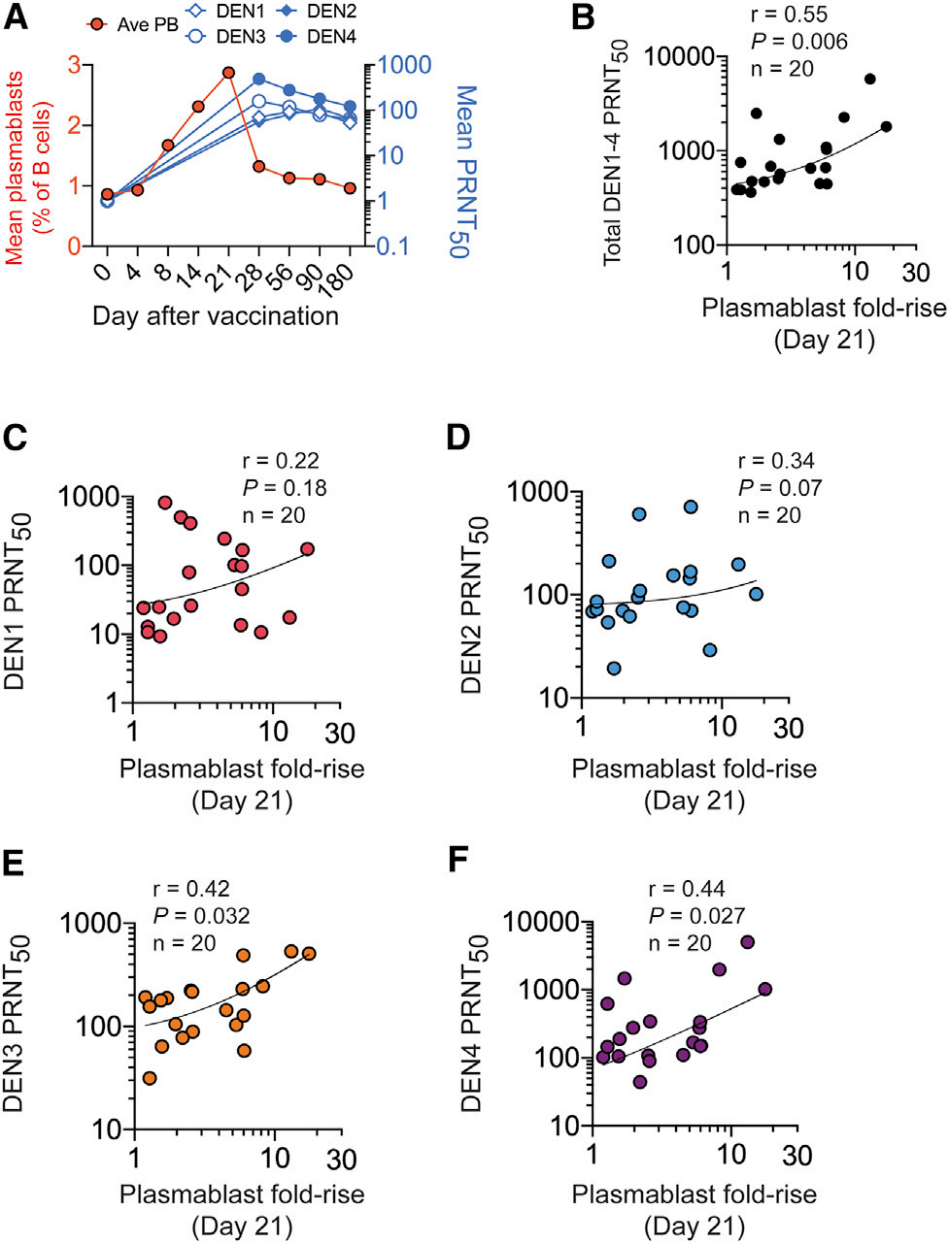
Induction of Plasmablasts and Serum Neutralizing Antibodies by TV003 (A) Kinetics of plasmablast (red, left y axis) and DENV1–DENV4 neutralizing antibody responses (right y axis, PRNT_50_) after vaccination with TV003. Mean responses in 21 subjects are shown. (B–F) Plasmablast responses (day 21 fold-rise) are plotted against the (B) sum of peak DENV1–DENV4 PRNT_50_ titers or against individual (C) DENV1, (D) DENV2, (E) DENV3, or (F) DENV4 peak PRNT_50_ values induced within 180 days after TV003. Spearman correlation coefficients and p values are reported. At least 2 technical replicates were performed for all PRNT_50_ measurements.

All TV003 vaccinees developed serum neutralizing antibodies to at least 3 DENV serotypes, and 92% developed a tetravalent response through day 180 after a single dose.^19^ Serum neutralizing antibodies to all 4 serotypes were present at day 28 post-vaccination and were maintained for at least 6 months (Figure 2A). We next determined whether the magnitude (frequencies and fold-rise) of the plasmablast response was correlated with DENV serum neutralization titers. We found a positive correlation (R = 0.55, p = 0.006) between day 21 plasmablast fold-rise and overall DENV1–DENV4 serum neutralizing antibody titers (Figure 2B) and with neutralizing antibodies to DENV3 and DENV4, while the relationship to DENV2 and DENV1 neutralizing antibodies was weak (Figures 2C–2F). Point-in-time plasmablast frequencies at days 14 or 21 after vaccination did not correlate with neutralizing antibodies (Figures S4A and S4B). Peak plasmablast induction (fold-rise at any point within 28 days after vaccination compared to baseline) exhibited a positive relationship with DENV3 neutralizing antibody titers and weaker positive associations with DENV3, -4, and overall DENV serum neutralizing antibody titers (Figure S4C). Thus, the magnitude of the plasmablast response to TV003 immunization correlated with peak DENV serum neutralizing antibody titers.

### TV003-Induced T Cell Response and Acute B Cell Response

Activated CD4^+^ T cells, particularly T follicular helper cells, can aid in the generation of broad antiviral antibody responses,^29^ prompting us to investigate whether TV003 induced CD4^+^ T cell activation in association with B cell responses. Although cytokine-independent assays have been used to detect such antigen-experienced cells in natural dengue,^30^ we directly assessed DENV-specific CD4^+^ T cells responses after TV003 vaccination by *ex vivo* stimulation of PBMCs with DENV-derived major histocompatibility class II peptide megapools as described.^31^, ^32^, ^33^ We previously showed that TV003 induced a robust DENV-specific T cell response in the same CIR287 study cohort, with the peak response on day 21 following immunization.^33^ For those subjects with matching CD4^+^ T cell, plasmablast, and neutralizing antibody data, we determined the fold-increase from baseline to day 21 for CD4^+^ T cell responses to be consistent with the day 21 plasmablast induction metric. We observed a positive, although not statistically significant trend of DENV-specific CD4^+^ T cell activation and plasmablast induction after TV003 vaccination (Figure S5A), but found a significant correlation (R = 0.86, p < 0.001) between DENV-specific interferon-γ^+^ (IFN-γ^+^) CD4^+^ T cell day 21 fold-rise and overall peak DENV neutralizing antibody titers (Figure S5B). These results implicate the acute CD4^+^ T cell response in the development of serum neutralizing antibody following TV003 immunization.

### Post-vaccination and Post-challenge Plasmablast Dynamics

**Figure 3:**
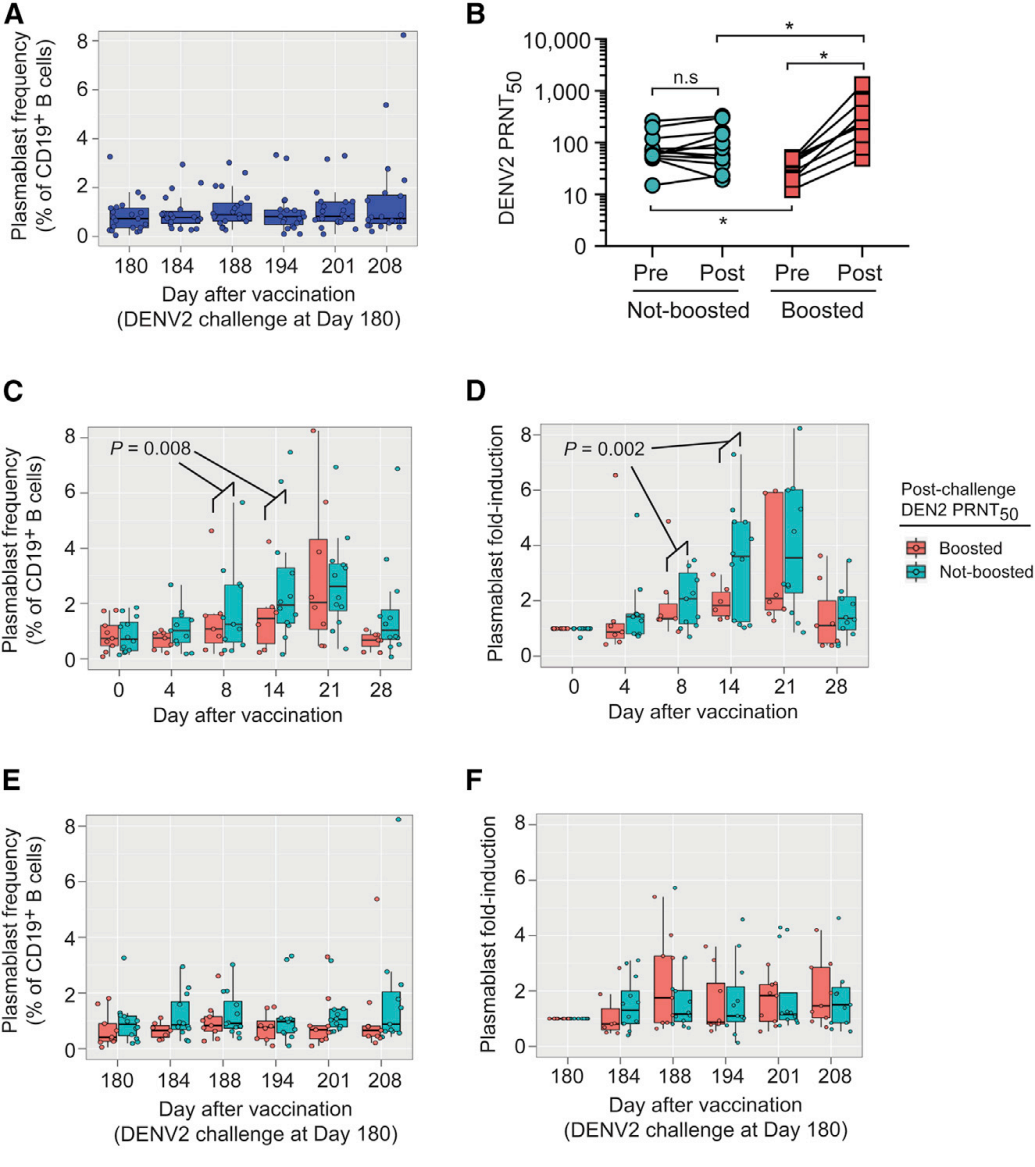
Post-TV003 and Post-challenge Plasmablast Induction Is Associated with Mode of Protection against Challenge with rDEN2Δ30. (A) Plasmablast frequencies were determined at intervals after DENV2 challenge (day 180) in subjects previously vaccinated with TV003 (n = 20). (B) DENV2 PRNT_50_ titers in TV003 vaccinees challenged with rDEN2Δ30 are plotted for day 180 after vaccination (i.e., at time of challenge) and at peak response after challenge. Subjects were classified as “boosted” or “non-boosted” depending on a ≥4-fold rise in DENV2 PRNT_50_ titers post-challenge compared to pre-challenge titers. (C and D) Plasmablast frequency (C) or (D) fold induction was assessed post-TV003 vaccination as a function of the subjects’ booster response. Data were fit to linear mixed-effects models, and a significant interaction between “boosted” and “day” on days 8 and 14 after vaccination was found by ANOVA analysis of the model containing both variables versus that with just the “boosted” term. (E and F) Plasmablast frequency (E) or (F) fold induction was assessed post-rDEN2Δ30 challenge as a function of the subjects’ booster response. At least 2 technical replicates were performed for all PRNT_50_ measurements. Boxplot areas show 25^th^-75^th^ percentiles with the median as a thick line and whiskers indicating the 95% confidence interval and closed circles indicate each data point from each subject.

Although plasmablasts were elicited after primary rDEN2Δ30 infection of flavivirus-naive subjects,^25^ we found that plasmablasts were not affected after rDEN2Δ30 challenge of subjects previously vaccinated with TV003 (Figure 3A). Compared to pre-challenge DENV2 antibody titers (i.e., at day 180 post-TV003) peak DENV2 serum neutralizing antibodies were boosted (≥4-fold increase) by DENV2 challenge in 9 of 21 protected vaccinees, but not in the other 12 protected vaccinees (non-boosted group) (Figure 3B). Plasmablast frequencies increased earlier and to a greater extent at days 8 and 14 post-immunization in subjects with sterile DENV2 protection (i.e., non-boosted group), compared to those with non-sterile DENV2 protection (i.e., boosted group) (Figure 3C). Similarly, plasmablast induction (i.e., fold-rise) was earlier and higher at days 8 and 14 after vaccination in the non-boosted subjects versus the boosted subjects (Figure 3D). Day 21 plasmablasts trended higher in non-boosted vaccinees but were not discriminatory for mode of protection (Figures 3C and 3D). We then assessed post-challenge plasmablasts in both groups of challenged TV003 vaccinees (boosted versus non-boosted), reasoning that the reactivation of TV003-elicited memory B cells by DENV2 challenge could generate plasmablast-like responses in boosted subjects. Post-challenge plasmablast frequencies and fold-induction were overall low and did not differ in magnitude or kinetics by mode of protection (Figures 3E and 3F). Our results suggested that early and robust plasmablast induction occurring at 1–2 weeks after TV003 vaccination was consistent with sterilizing humoral immunity to subsequent DENV2 challenge.

### Breadth of DENV-Specific Memory B Cells Induced by TV003 Immunization

A key goal of live attenuated tetravalent dengue vaccines is the generation of durable memory. To determine whether TV003 elicited a durable DENV-specific memory B cell (MBC) response, we used genetic reprogramming^34^ to immortalize class-switched (IgM^−^) memory (CD27^+^) CD19^+^ B cells from TV003 vaccinees at 6 months after immunization and screened their secreted IgG for reactivity to DENV virions. We screened MBCs for DENV2 reactivity because this was a tetravalent vaccine/DENV2 challenge study, and this would allow us to compare DENV2-specific MBC frequencies to our previous study that estimated DENV2-binding MBC frequencies after primary rDEN2Δ30 infection.^25^ MBC transduction efficiency (as indicated by GFP transduction marker positivity) averaged 67% (range 26%–94%; Table S2) across the 11 subjects, indicating broad repertoire coverage. To reduce the complexity of the transduced polyclonal MBC population, we cultured GFP^+^ MBC at 50 cells/well and screened for DENV2-reactive immunoglobulin G (IgG) by ELISA. We estimated the frequencies of DENV2-reactive cells in the MBC pool by dividing the number of DENV-binding-positive wells by the total number of GFP^+^ cells screened as we have done previously.^25^^,^^35^

**Figure 4:**
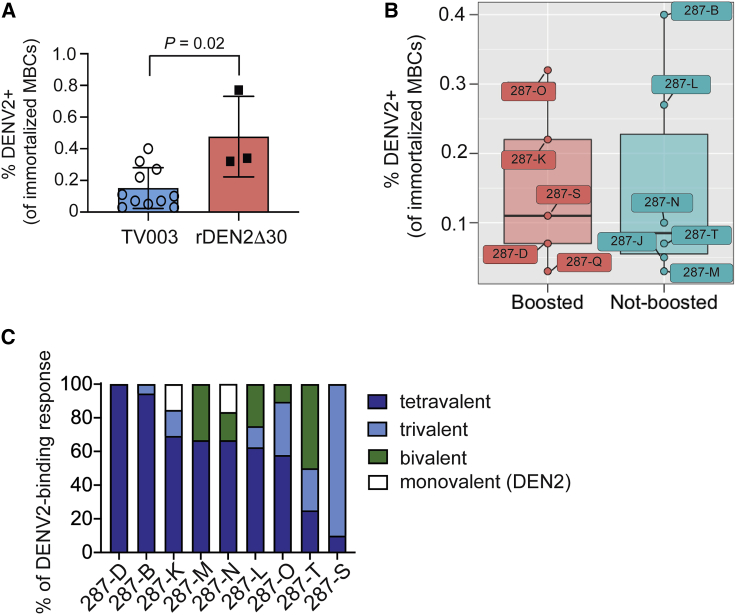
TV003 Vaccination Induces DENV-Specific Memory B Cells (MBCs) (A) IgM^−^CD27^+^ MBCs were isolated 6 months after TV003 immunization (n = 11 subjects) or after primary DENV2 infection (n = 3 subjects), immortalized with BCL6 + Bcl-x_L_ (6XL), and supernatants from polyclonal (50 cells/well) cultures of 6XL-immortalized cells were screened for IgG binding to DENV2 (the challenge virus). The frequency of DENV2-reactive MBC was estimated from the total number of cells screened and based on the average of 1–2 reactive clones in a positive polyclonal culture, as previously shown.^35^ Statistical significance was determined using the non-parametric Mann-Whitney test. Bar graphs indicate the mean with error bars representing standard deviation across all individual subjects (open circles for TV003 vaccinees and closed squares for rDEN2Δ30-infected subjects). (B) Frequency of DENV-specific MBC at time of DENV2 challenge (day 180) in TV003 vaccinees with non-sterile protection (i.e., boosted post-challenge DENV2 neutralizing antibody levels) and those with sterile protection from DENV2 infection (i.e., non-boosted DENV2 antibody levels). Boxplot areas show 25^th^-75^th^ percentiles with the median as a thick line and whiskers indicating the 95% confidence interval and closed circles indicate each data point from each subject (labeled with de-identified study identifiers). (C) DENV2-reactive MBC cultures from 9 TV003-vaccinated subjects (subject IDs on x axis) were screened individually for binding to DENV1, DENV3, and DENV4 virions by ELISA and the proportions of monovalent (DENV2-only); bivalent (DENV2 + 1 other DENV); trivalent (DENV2 + 2 other DENVs); and tetravalent (DENV1–DENV4) are expressed as a percentage of the total response. At least 2 technical replicates were performed for all ELISA-binding measurements.

We estimated an average 0.15% of the MBC repertoire to be DENV2 reactive (range 0.03%–0.40%) at 6 months post-TV003 vaccination (Figure 4A). This frequency was lower than that found for primary rDEN2Δ30-infected subjects at the same time point (Figure 4A).^25^ Among the TV003 group, DENV2-specific MBC frequencies did not discriminate between subjects that subsequently did or did not boost DENV2 serum neutralizing antibody levels after challenge (Figure 4B). We also assessed whether the endpoint of post-vaccination DENV2-specific MBC frequencies were reflective of the vaccine-induced plasmablast response or serum antibody titers. We did not find significant correlations for day 180 DENV2-specific MBC frequencies with post-TV003 plasmablasts at days 14 or 21 or overall serum neutralizing antibodies (Table S3). We therefore conclude that TV003 induces DENV2-reactive MBCs at 6 months after TV003 vaccination, but this metric was not sufficient to predict sterile immunity from DENV2 challenge in a subset of TV003 vaccinees.

Humoral immunity to DENV is a complex mixture of serum antibodies that bind to DENV in a serotype-specific (also called type specific [TS]) or cross-reactive (CR) manner. We next asked the extent to which DENV-reactive IgG elicited by TV003 vaccination was TS or CR at the cellular level (i.e., MBCs). Since this was a TV003/DENV2 challenge study, we screened the DENV2-binding MBC-derived IgGs for binding to DENV1, DENV3, and DENV4 antigens. Among the MBCs that were immortalized, we found a broad range of responses from DENV2-TS to bi-, tri-, and tetravalent responses in TV003 vaccinees (Figure 4C). Our results demonstrate that TV003 induces a broad array of DENV2-reactive MBCs that are readily found 6 months after vaccination.

### Multivalent Serum-Neutralizing Antibody Responses Induced by TV003

**Figure 5:**
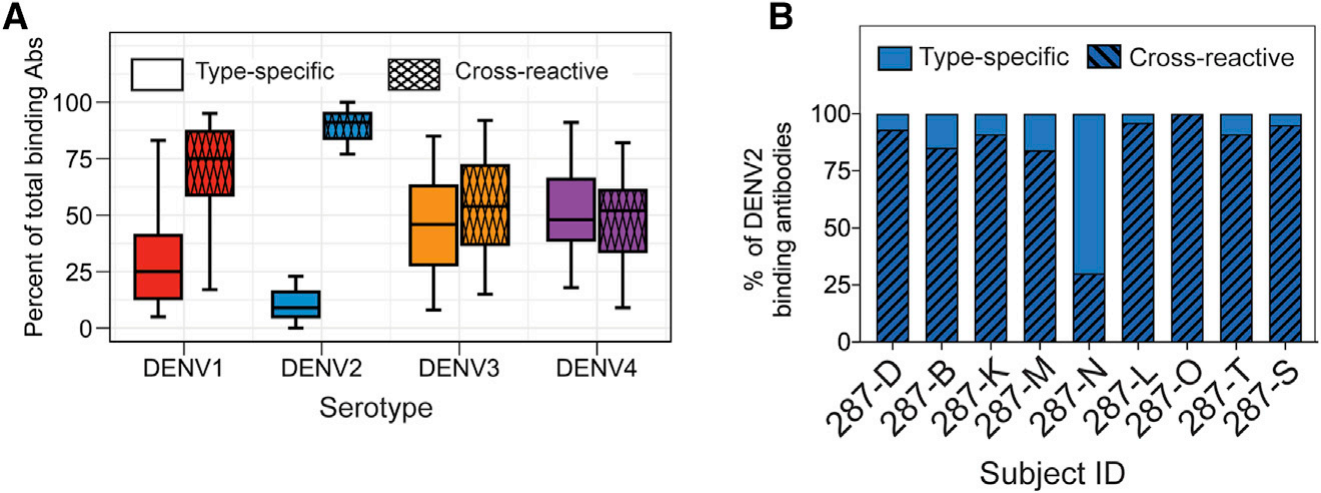
Properties of the DENV-Reactive Serum Antibody Response in Flavivirus-Naive Subjects That Received the Live Attenuated Tetravalent DENV Vaccine TV003 The properties of the serum DENV-binding response in convalescent (6 months after vaccination) TV003 vaccinees was determined by virus depletion and DENV-binding ELISAs. (A) Type-specific and cross-reactive binding to each serotype was determined. Boxplots represent the 25th–75th percentiles with whiskers showing the 5th–95th percentiles, with the horizontal bar showing the median of the fraction of each type of antibody measured across subjects (n = 21 subjects). (B) The proportions of DENV2-reactive serum antibodies were classified as type specific (open bars) and cross-reactive (hatched bars) was determined for a subset of 9 subjects with memory B cell reactivity data (see Figure 4). At least 2 technical replicates were performed for all ELISA-binding measurements.

To qualitatively explore the composition of total TS and CR binding antibodies for each DENV serotype at 6 months post-TV003, we used an established virus depletion approach.^36^, ^37^, ^38^ To assess DENV2-TS and -CR binding activity, we depleted TV003 vaccine serum samples with beads coated with DENV1, DENV3, and DENV4 antigens (heterotypic depletion). For other serotypes, we incubated serum with beads coated with DENV2 (homotypic depletion). For all of the serotypes, we found that between 50% and 80% of total binding activity was due to CR antibodies (Figure 5A). In agreement with our MBC results for DENV2, we found a broad span of serum DENV2 binding activity with TS:CR ratios ranging from 0 to 0.7 (Figure 5B).

Overall, our results demonstrated that TV003 elicited an early plasmablast response whose kinetics appeared to correlate with subject-specific modes of protection from DENV2 challenge. TV003 also induced a tetravalent response in both serum and MBCs, suggesting durable immunity across multiple humoral compartments.

## Discussion

Here, we have examined the cellular underpinnings of the B cell response over time in relation to vaccine viremia and serum antibodies in the context of TV003-mediated protection from viremia upon challenge with rDEN2Δ30. We and others have found a positive relationship between plasmablast induction and viremia during acute dengue infection.^22^^,^^25^ We extended this relationship to immunization with the live attenuated vaccine TV003. Whereas primary rDEN2Δ30 infection led to peak viremia titers of 2–3 log_10_ plaque-forming units (PFUs)/mL and a peak plasmablast frequency of 2%–20% of B cells on day 14 after infection,^25^ the more attenuated TV003 generated a lower peak viremia of 0.5–1.7 log_10_ PFU/mL, lower plasmablast peak frequencies (∼2%–7% of B cells), and at a later time point (days 14–21) after vaccination. These results suggest that in the settings of both primary DENV infection or vaccination with a live attenuated vaccine, viral load drives the magnitude and kinetics of plasmablast induction, predominantly occurring within 1 month after vaccination. Occasionally, high plasmablast levels were detected outside the day 0–28 window in 2 subjects (287-G and 287-H; Figure S1A). We do not know whether these were DENV-specific cells, but we posit that these cells were not driven by vaccine virus due to clearance of viremia by this point. The magnitude and kinetics of plasmablast induction by TV003 were similar to those following immunization with the efficacious yellow fever virus vaccine YF-17D.^22^^,^^39^ A recent report on the plasmablast response following TV003 immunization in a DENV-experienced subject showed a massive 70-fold plasmablast increase,^40^ possibly the manifestation of an anamnestic response following immunization. We previously showed that TV003 vaccination generated higher DENV serum neutralizing antibodies in subjects who were flavivirus experienced at the time of vaccination compared to those who were flavivirus naive.^13^ Future work will determine whether flavivirus experience influences the plasmablast response to TV003 vaccination. We did not observe a secondary plasmablast response in rDEN2Δ30-challenged TV003 vaccinees. Given the robust protection against viremia afforded by TV003 vaccination, it is possible that there was too little challenge virus antigen to activate CD14^+^CD16^+^ monocytes to stimulate plasmablasts in the vaccine/challenge setting.^41^

DENV-specific plasmablasts are highly induced in natural dengue infection.^22^^,^^28^ We recently isolated >40 DENV2-binding monoclonal antibodies—several of which were neutralizing—from plasmablasts induced by rDEN2Δ30 infection.^25^ We found here that plasmablasts correlated also with TV003 serum neutralizing antibodies. Since plasmablast responses were concordant with both viremia and antibodies, our results suggested that these cells may play an important mechanistic cellular link between replication of live attenuated flavivirus vaccines such as TV003 and the induction of neutralizing antibody responses.

To evaluate whether early B cell responses may explain TV003-associated modes of protection, we examined plasmablast induction in subjects experiencing sterile and non-sterile protection from rDEN2Δ30 challenge. An earlier and more robust TV003-induced plasmablast response was associated with higher DENV2 antibody titers at the time of rDEN2Δ30 challenge and provided sterilizing immunity against rDEN2Δ30. Meanwhile a weaker TV003-induced plasmablast response was associated with non-sterilizing immunity upon rDEN2Δ30 challenge. We posit that an earlier and stronger vaccine-induced plasmablast expansion may generate a higher neutralizing antibody baseline in subjects experiencing sterilizing immunity, compared to a weaker vaccine-induced plasmablast response in subjects with non-sterilizing immunity. In addition, there may be subject-specific clonal repertoire differences among DENV2-binding B cells that produce highly potent TS neutralizing antibodies such as 2D22^42^^,^^43^ or broadly CR neutralizing antibodies such as those that target E-dimer epitopes.^27^

Our data showed that TV003 induced antigen-specific MBCs at 6 months post-vaccination. This finding extends work by us and others showing the presence of antigen-specific MBCs after natural infection with DENV,^25^^,^^44^, ^45^, ^46^ DENV4 monovalent vaccination,^47^ Zika virus infection,^35^ hepatitis virus C infection,^48^^,^^49^ or respiratory syncytial virus exposure.^34^ Furthermore, by comparing the proportion of DENV2-binding MBCs at 6 months after primary DENV2 infection and TV003 vaccination, our results suggest that early viremia after infection or vaccination may help to “set” antigen-specific MBC frequencies in early convalescence.

Given that TV003 induces durable MBCs but distinct post-challenge DENV2 antibody responses, we hypothesized that secondary exposure to rDEN2Δ30 antigens would recall TV003-elicited MBCs into a germinal center-like phenotype that may produce plasmablasts. However, secondary plasmablasts were not observed after challenge. One potential explanation is the lack of rDEN2Δ30 virus replication (or genome) in the serum after challenge of TV003 vaccinees may translate to a lack of antigenic stimulation for a plasmablast response. Another possibility is that there may be limited clonal overlap between plasmablast and MBC repertoires, as seen previously for DENV.^25^^,^^50^ To address this possibility, we also performed a correlation analysis of post-vaccination DENV2-binding MBC frequencies and plasmablasts and serum neutralizing antibodies after vaccination and failed to find statistically significant relationships. These data suggested that post-vaccination precursor MBC frequencies were not related to the boost response in a subset of rDEN2Δ30-challenged TV003 vaccinees. In agreement with our prior findings,^25^ others have shown that the MBC repertoire has limited overlap with plasmablasts.^51^ Moreover Ellebedy et al.^51^ showed that MBCs exhibited substantial overlap with the CD71^+^ “activated B cell” (ABC) response, which occurs later than the plasmablast response. Although we did not capture the ABC response in our staining panel, it is possible that rDEN2Δ30 challenge re-activated quiescent vaccine-associated MBCs to assume an ABC-like phenotype and contribute to the “boost” response.

Vaccination with TV003 elicits neutralizing antibodies against all 4 DENV serotypes^15^, ^16^, ^17^^,^^19^ and protection against rDEN2Δ30 challenge,^19^ but the specificities of the DENV-specific antibody responses are unclear. In the serum of TV003 vaccinees, the DENV2 binding response was skewed toward CR antibodies, which may be reflective of the higher stoichiometry of conserved versus serotype-specific epitopes present in a tetravalent vaccine. In line with this, previous work has shown that for DENV infection or monovalent vaccination, TS-binding MBC clones are rarer than CR clones.^44^^,^^47^ Our results for MBCs were congruent with this finding, since we observed fewer DENV2-TS compared to CR MBCs at 6 months post-TV003 vaccination. This is akin to finding that the CD8^+^ T cell response to TV003 vaccination is highly focused on epitopes conserved across all four DENV serotypes.^52^

In dissecting the serum response to DENV natural infection^53^^,^^54^ and monovalent or tetravalent DENV vaccination, we found that TS antibodies comprise a substantial proportion of the serum neutralizing response.^36^, ^37^, ^38^ We also note that broadly neutralizing CR antibodies have been isolated from individuals with multiple DENV exposures.^27^ It is formally possible that some CR neutralizing antibodies could be eliminated our heterologous depletion strategy in serum. Functional analysis of MBC-derived DENV2-reactive antibodies was hampered by insufficient IgG to perform neutralization assays. Furthermore, we recovered a relatively low number of DENV2-reactive MBCs, presumably due to the limited replication potential of the inoculum. Nonetheless, our MBC studies show that TV003 elicits a broad panel of virus-specific MBCs consisting of TS and CR antibodies that are durable at 6 months after vaccination.

Our DENV2-focused screening strategy was not able to investigate TS and CR responses for the other serotypes. Although we readily detected DENV-specific MBCs at 6 months after TV003 vaccination, it will also be important to determine how long DENV-specific B cell clones persist beyond 6 months and whether this is akin to tetanus- or smallpox-reactive B cells, which have been shown to persist for decades.^55^^,^^56^

In addition to humoral immunity, we and others have found that durable CD4^+^ and CD8^+^ T cell responses are induced by tetravalent live attenuated dengue vaccination.^32^^,^^33^^,^^52^ Thus, it is also possible that T cells activated by TV003 protected against rDEN2Δ30 challenge in concert with B cells in subjects exhibiting sterilizing humoral immunity. Our data indicated a positive relationship between DENV-specific CD4^+^ T cell response and serum DENV neutralizing antibody titers following TV003 immunization.

In summary, early plasmablast responses to TV003 vaccination correlate with sterilizing humoral immunity to rDEN2Δ30 challenge. Maintenance of durable balanced activity against all DENV serotypes elicited by TV003 may involve both MBCs and plasma cells producing potently neutralizing TS and CR antibodies. We posit that analyzing the different cellular components of the humoral response to vaccination may provide additional markers by which to assess vaccine immunogenicity and performance.

### Limitations of Study

A limitation of this study is that the dengue vaccine studied here is experimental and has not completed efficacy trials and has not been approved for prevention of dengue disease. While all subjects were screened for previous flavivirus exposure before vaccination, exposure to other infections before or during the study could affect patient-specific immune dynamics independently of the vaccine-induced response. Moreover, the rDENV2Δ30 challenge may not reflect infection with wild-type virus. The potential links between adaptive immune activation after TV003 vaccination and protection from challenge raised by our study will need to be further validated in clinical settings, including endemic exposures and in studies with a larger sample size.

## STAR★Methods

### Key Resources Table

**Table:** 

REAGENT or RESOURCE	SOURCE	IDENTIFIER
**Antibodies**		

Goat Anti-Human IgG (Fc specific), highly cross adsorbed-Alkaline Phosphatase	Sigma Millipore	Cat. #SAB3701277
4G2 (anti-flavivirus E)	Stephen Whitehead	Cat. #ATCC HB-112
anti-human CD3 (UCHT1) FITC-conjugated	BioLegend	Cat. #300406
anti-human CD3 (UCHT1) Pacific Blue-conjugated	BioLegend	Cat. #300431
anti-human CD3 (UCHT1) AlexaFlour700-conjugated	BD Biosciences	Cat. #557943
anti-human CD4 (OKT4, BV510-conjugated	BioLegend	Cat. #317444
anti-human CD14 (MΦPg) BUV395-conjugated	BD Biosciences	Cat. # 563561
anti-human CD14 (HCD14) Pacific Blue-conjugated	BioLegend	Cat. #325616
anti-human CD19 (HIB19) PE-Dazzle 594-conjugated	BioLegend	Cat. #302252
anti-human CD19 (SJ25C1) BUV395	BD Biosciences	Cat. # 563549
anti-human CD20 (2H7) PE-Cy7-conjugated	BioLegend	Cat. # 302311
anti-human CD22 microbeads	Miltenyi	Cat. # 130-046-401
anti-human CD27 (O323) BrilliantViolet510-conjugated	BioLegend	Cat. #302835
anti-human CD27 (O323) PE-Cy7-conjugated	BioLegend	Cat. #302838
anti-human CD38 (HIT2) AlexaFluor 647-conjugated	BioLegend	Cat. #303514
anti-human IgM (MHM-88, PerCP-Cy5.5)	BioLegend	Cat. #314512
anti-human IFN-γ (4S.B3) FITC	eBioscience	Cat. #11-7319-41

**Bacterial and Virus Strains**		

Dengue virus serotype 1 (West Pacific 74) WHO reference strain	Stephen Whitehead, National Institute of Allergy and Infectious Disease (NIAID)	GenBank AY145121
Dengue virus serotype 2 (New Guinea C)	Stephen Whitehead, NIAID	GenBank AF038403.1
Dengue virus serotype 2 (strain S-16803) WHO reference strain	Aravinda De Silva, University of North Carolina-Chapel Hill (UNC)	GenBank GU289914
Dengue virus serotype 3 (Sleman/78)	Stephen Whitehead, NIAID	GenBank AY656169
Dengue virus serotype 3 (CH53489) WHO reference strain	Aravinda De Silva, UNC	GenBank DQ863638
Dengue virus serotype 4 Dominica/81	Stephen Whitehead, NIAID	GenBank AY648301
Dengue virus serotype 4 (TVP-376) WHO reference strain	Aravinda De Silva, UNC	GenBank KC963424

**Biological Samples**		

Human Serum	University of Vermont Vaccine Testing Center and Johns Hopkins Center for Immunization Research	Clinicaltrials.gov identifiers: NCT01072786, NCT02021968
Peripheral blood mononuclear cells (PBMC)	University of Vermont Vaccine Testing Center and Johns Hopkins Center for Immunization Research	Clinicaltrials.gov identifiers: NCT01072786, NCT02021968

**Chemicals, Peptides, and Recombinant Proteins**		

recombinant human IL-21	Peprotech	Cat. #200-21
p-Nitrophenyl phosphate	Sigma	Cat. #N2770
Retronectin	Takara	Cat. #T202
Polybead Microspheres	Polysciences Inc,	Cat. #17135-5
Dynabeads M-280, Tosylactivated	ThermoFisher	Cat. #14204
Ionomycin	Millipore Sigma	Cat. # I3909-1ML; CAS: 56092-81-0
Phorbol 12-myristate 13-acetate	Millipore Sigma	Cat. #P8139; CAS: 16561-29-8
GolgiPlug	BD Biosciences	Cat. #555029
DENV-specific MHC class II peptide megapool	A&A, San Diego, CA	http://www.iedb.org, ^33^
Dimethyl sulfoxide	Millipore Sigma	Cat. # D2650; CAS: 67-68-5
LIVE/DEAD Fixable Blue Dead Cell Stain Kit, for UV excitation	ThermoFisher	Cat. #L34962
4’,6-Diamidino-2-Phenylindole, Dihydrochloride (DAPI)	ThermoFisher	Cat. #D1306; CAS: 28718-90-3

**Experimental Models: Cell Lines**		

African green monkey kidney cells (Vero-81)	Stephen Whitehead, NIAID	RRID:CVC_0059
Stable CD40L-L cells	Hergen Spits, U. Amsterdam	N/A

**Recombinant DNA**		

LZRS-BCL6-T2A-BCLXL-IRES-GFP	Diehl Lab	N/A

**Software and Algorithms**		

Prism 8.4.3	Graphpad, Inc.	N/A
R 3.6.1	R Group	http://cran.wustl.edu/bin/macosx/
FlowJo 10	Treestar	N/A

### Resource Availability

#### Lead Contact

Further information and requests for resources and reagents should be directed to and will be fulfilled by the Lead Contact Author Sean Diehl (sean.diehl@med.uvm.edu).

#### Materials Availability

No new unique reagents were generated in this study.

#### Data and Code Availability

The published article includes all datasets generated or analyzed during this study.

### Experimental Model and Subject Details

#### Ethics statement

Subjects in this study were participants of phase I studies to evaluate the safety, immunogenicity (trial CIR268, Clinicaltrials.gov NCT01072786^15^) and experimental challenge efficacy (trial CIR287, Clinicaltrials.gov NCT02021968^19^) of the tetravalent live attenuated dengue vaccine TV003. Samples were de-identified by assignment of A-Z identifiers. All subjects were serologically confirmed as flavivirus-naive at the time of immunization. Studies were approved by the Institutional Review Boards at the University of Vermont and the Western Institutional Review Board (Johns Hopkins University). Informed consent was obtained in accordance with federal and international regulations (21CFR50 and ICHE6). External monitoring was performed by the National Institute of Allergy and Infectious Diseases Data Safety Monitoring board every 6 months.

#### Viruses

DENV1 West Pacific 74, DENV2 New Guinea C, DENV3 Sleman/78, and DENV4 Dominica/81 were propagated in Vero-81 cells (American Type Culture Collection; CCL81, RRID:CVC 0059). These viruses were used both in DENV binding ELISA and neutralization tests. Titers of virus stocks were determined by serial dilution of stocks and infection of Vero-81 cell monolayers on 24 well plates. Optimal dilution for use in ELISA was determined by serial dilution of stocks in DENV binding ELISA (see below) using a DENV cross-reactive mAb 1M7^57^) at 1 μg per well to achieve an OD_405_ = 1.0 for each individual serotype to normalize among serotypes and assays. For the depletion assays, the Vero-81 derived purified WHO references strains, DENV1 (American genotype; strain West Pac74), DENV2 (Asian genotype; strain S-16803), DENV3 (Asian genotype; strain CH-53489), and DENV4 (American genotype; strain TVP-376) were used as described previously.^36^

#### Cells

Vero-81 cells were obtained from Stephen Whitehead and maintained in Opti-MEM I Reduced Serum Medium, no phenol red (ThermoFisher cat. 11058021) supplemented with 10% fetal bovine serum (FBS).

### Method Details

#### Clinical sample procurement

At study visits, blood was collected by venipuncture into serum separator tubes for analyses of viremia and serology, and into EDTA tubes for isolation of peripheral blood mononuclear cells (PBMC). Serum was frozen at –20°C until use. PBMC were isolated by Ficoll-paque density gradient separation, counted, and frozen in cell culture medium with 10% dimethyl sulfoxide (DMSO) and 40% fetal bovine serum (FBS), and cryopreserved in liquid nitrogen vapor phase.

#### Serological analyses

Sera collected every other day up to day 16 following TV003 immunization and again on this scheme after DENV2 challenge were tested for infectious virus by titration and infection of Vero-81 cells. Viral plaques were detected with serotype-specific monoclonal antibodies as previously described.^32^ Serum neutralizing antibody titers against DENV1-4 were determined by plaque reduction neutralization test (PRNT), using lowest serum dilution that yielded a 50% reduction in viral plaques (PRNT_50_) as previously described.^19^

#### Plasmablast phenotyping

PBMC were surface-stained with the following fluorophore-conjugated monoclonal antibodies: anti-CD19 (HIB19, PE-Dazzle 594), anti-CD3 (UCHT1, Pacific Blue), anti-CD14 (HCD14, Pacific Blue), anti-CD20 (2H7, PE-Cy7), anti-CD27 (O323, BrilliantViolet 510), anti-CD38 (HIT2, AlexaFluor 647), all from BioLegend. 4’,6-diamidino-2-phenylindole (DAPI, Invitrogen) was used at 3 μM in staining buffer as a viability dye. Data were acquired on a BD LSRII (BD BioSciences using BD FACS DIVa software. Plasmablasts were defined from lymphocyte forward × side scatter-A and DAPI– gating as CD3^–^CD14^–^CD19^+^CD20^low/–^CD27^+^CD38^hi^ cells using FlowJo version 10 (TreeStar).

#### Memory B cell isolation and immortalization

B cells were enriched from PBMC using positive magnetic selection with microbead-conjugated anti-CD22 antibodies (Miltenyi). CD22-enriched B cells were labeled with fluorophore-conjugated monoclonal antibodies anti-CD3 (UCHT1, FITC), anti-CD19 (HIB19, PE-Dazzle 594), anti-CD27 (O323, PE-Cy7) and anti-IgM (MHM-88, PerCP-Cy5.5), and DAPI (Invitrogen). All flow cytometry antibodies were purchased from Biolegend. CD3^–^CD19^+^IgM^–^CD27^+^ memory B cells (MBC) were purified by fluorescence-activated cell sorting (FACS) on a FACSAriaIII using the BD FACSDiva software from live (DAPI–) lymphocyte singlets and deposited into complete Iscove’s Modified Dulbecco’s Medium (IMDM) supplemented with 8% FBS (Atlanta Biologicals), 100 units/mL penicillin (GIBCO), and 100 μg/mL streptomycin (GIBCO).

Purified MBC were immortalized with BCL-6 and Bcl-xL via retroviral transduction as previously described.^25^^,^^34^ MBC were first activated with 1 × 10^5^ irradiated (50 Gy) human CD40L-expressing mouse fibroblasts and 50 ng/mL recombinant human interleukin 21 (rhIL-21, Peprotech) on tissue culture-treated 24-well plate for 36-48 hours at 37°C, 5% CO_2_. Following activation, cells were suspended in 0.25 mL serum-free IMDM and mixed with equal volume of retrovirus. Cells/virus mixture was added to a non-tissue culture-treated 24-well plate coated with 30 ng/mL retronectin (Takara, catalog no. T202) and blocked with 2% human serum albumin in phosphate buffer saline. The plate was centrifuged at room temperature for 1 hour at 700 × g, followed by incubation at 37°C, 5% CO_2_ for 6 hours to overnight. Cells were then washed and maintained in complete IMDM with rhIL-21 and CD40L cells in a tissue culture-treated 24-well plate. After approximately two weeks of culture, MBC expressing CD19 and GFP (a marker for transduction) were sorted into polyclonal cultures at 50 cells/well onto 96 well plates containing completed IMDM supplemented with rhIL-21 and 2.5 × 10^4^ CD40L cells. Polyclonal MBC cultures were maintained for three weeks before screening supernatants for DENV IgG reactivity.

#### ELISA to detect DENV-specific antibodies

The assays were performed using microplates (ThermoFisher, catalog no. 44-2404-21) as previously described.^25^ Briefly, virus was captured by plate-adsorbed mouse cross-reactive anti-DENV envelope (E) protein monoclonal antibody 4G2. IgG-containing polyclonal supernatant was then added and positive DENV binding was detected by alkaline phosphatase-conjugated goat anti-human IgG (Fc) antibody (Millipore Sigma) and p-nitrophenyl phosphate substrate (Millipore Sigma). Reaction color change, indicating DENV-binding, was measured by spectrophotometry as OD_405_.

#### Antibody depletion from immune sera

Depletion of cross-reactive (CR) or type-specific (TS) antibodies from TV003 immune sera was performed as described previously.^36^ Purified DENV was adsorbed onto microbeads (Polybead Microspheres, Polysciences Inc or Dynabeads M-280 Tosylactivated, ThermoFisher). Beads adsorbed with bovine serum albumin (BSA) were used as a control. Human sera were diluted 1:10 in 1 × PBS and incubated with BSA- or virus-adsorbed beads for 1 h at 37°C with end-over-end mixing. Depleted sera were then tested for binding to the target serotype by ELISA as described above. For estimating the relative amount of DENV2 TS antibodies, beads incubated with a heterologous mixture containing an equivalent amount of DENV1,3,4 antigens were used. To estimate the % TS antibodies against DENV1, DENV3, or DENV4 beads incubated with DENV2 antigen were used. The percentage of CR and TS IgGs against each serotype were calculated using the following formulas:

$$\%\mathrm{CR}\;\mathrm{binding}\;\mathrm{Abs}=\frac{\Delta ELISA\;OD\;after\;hetero\log ous\;serotype\;depletion\;versus\;BSA\;control}{\Delta ELISA\;OD\;after\;hetero\log ous\;serotype\;depletion\;versus\;BSA\;contro}\times 100\%$$

%TSbindingAbs=100%−%CRbindingAbs

#### T cell analyses

*Ex vivo* IFN-γ responses in CD4^+^ T cells were determined by stimulation of PBMCs from TV003 vaccinees (CIR287 trial) at various times after vaccination with 12-14 aa DENV peptide megapools and intracellular flow cytometric analysis as described.^33^ Each sample included phorbol 12-myristate 13-acetate A, (100 ng/mL, Millipore Sigma) and ionomycin (1 μg/mL, Millipore Sigma) stimulation or incubation with DMSO (Millipore Sigma) as positive and negative controls, respectively. After stimulation for 2 hr at 37°C, GolgiPlug (BD Biosciences) was added and cells incubated for an additional 4 hr at 37°C. After stimulation cells were stained with anti-human CD3 (UCHT1, AlexaFlour700, BioLegend), anti-human CD4 (OKT4, BV510), anti-human CD14 (MΦPg, BUV395, BD Biosciences), anti-human CD19 (SJ25C1, BUV395, BD Biosciences), anti-human CD45RA (HI100, eFlour 450, ThermoFisher), anti-human CD197/CCR7 (G043H7, PerCP-Cy5.5, BioLegend), anti-human IFN-γ (4S.B3, FITC, eBioscience), and LIVE/DEAD Fixable Blue (ThermoFisher). Data were collected on a BD LSRII and Live/Dead Blue^–^CD3^–^CD14^–^CD19^–^ CD3^+^CD4^+^ IFN-γ^+^ cells were analyzed using FlowJo version 10 (TreeStar). Responses were expressed as the ratio of IFN-γ^+^ CD4^+^ T cell frequencies (out of total CD4^+^ T cells) at baseline versus at day 21 after TV003 in each subject.

### Quantification and Statistical Analysis

Differences in plasmablast or CD19^+^ B cell levels at different time points were assessed using ANOVA and the post hoc Tukey’s Honestly Significantly Different (HSD) test for multiple comparisons in R (version 3.6.1). Correlation analyses between DENV viremia, plasmablasts, dengue-specific IFN-γ^+^CD4^+^ T cells, and DENV serum neutralizing antibody titers were performed using nonparametric Spearman correlation tests using GraphPad Prism (Version 8.4.3). To determine if/how the pattern of plasmablast induction post-vaccination or post challenge was related the mode of protection (boosted = nonsterile versus not-boosted = sterile) over multiple time points we used a linear mixed effects (LME) model^58^ that allows for parametric repeated-measures testing incorporating error to account for data limitations such as sporadic missing samples (due to missed subject visit or sample viability). This was done using the lme R package was used in RStudio (R 3.6.1). ANOVA testing was then performed on LME models containing “boosting” and “day” versus “boosting” alone as interaction terms to evaluate significant difference in the model outputs. *P*-values are indicated.

## Supplemental Information

Document S1. Figures S1–S5 and Tables S1–S3

Document S2. Article plus Supplemental Information
