# Supplementary material for: Stimulation of B Cell Immunity in Flavivirus-Naive Individuals by the Tetravalent Live Attenuated Dengue Vaccine TV003
Source: Cell Rep Med. 2020 Dec 22;1(9):100155. doi: 10.1016/j.xcrm.2020.100155 (PMC7762770; doi:10.1016/j.xcrm.2020.100155)
Supplement: Document S1. Figures S1–S5 and Tables S1–S3 [file mmc1.pdf]

Cell Reports Medicine, Volume *1*

## Supplemental Information

# Stimulation of B Cell Immunity in Flavivirus-Naive Individuals by the Tetravalent Live Attenuated Dengue Vaccine TV003

**Huy A. Tu, Usha K. Nivarthi, Nancy R. Graham, Philip Eisenhauer, Matthew J. Delacruz, Kristen K. Pierce, Stephen S. Whitehead, Jonathan E. Boyson, Jason W. Botten, Beth D. Kirkpatrick, Anna P. Durbin, Aravinda M. deSilva, and Sean A. Diehl**

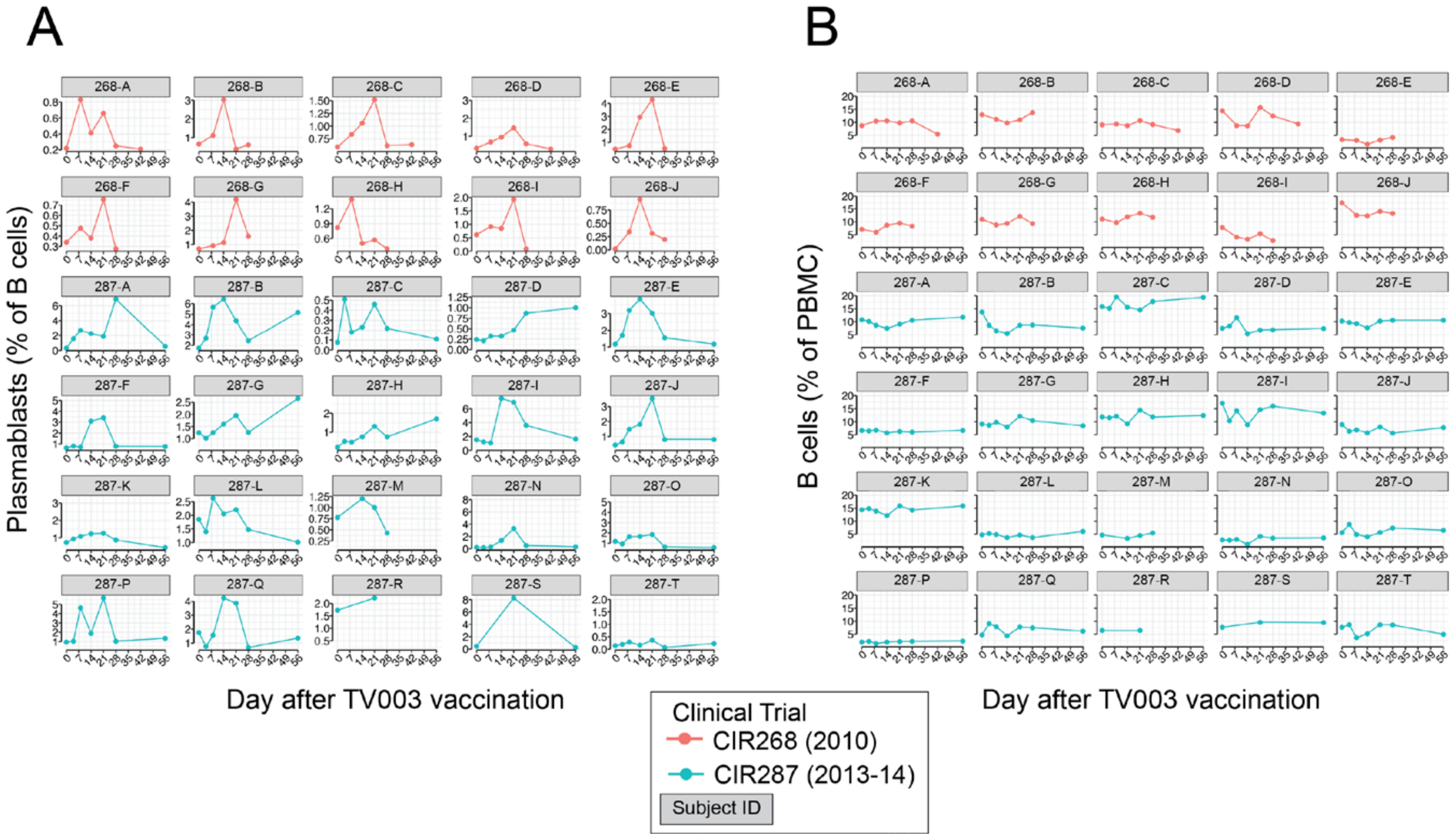


**Supplemental Fig. S1 | Patient-specific plasmablast and B cell frequencies after TV003 vaccination.** The frequency of (**A**) $CD19^{+}CD20^{low/-}CD38^{hi}CD27^{hi}$ plasmablasts and (**B**) total $CD19^{+}$ B cell levels after vaccination with the tetravalent live attenuated dengue vaccine TV003. Coded subject IDs with “268-A…J” refer to the 2010 CIR268 trial and those with “287-A…T” are from the 2013-14 CIR287 vaccine/challenge trial. Related to Figure 1.

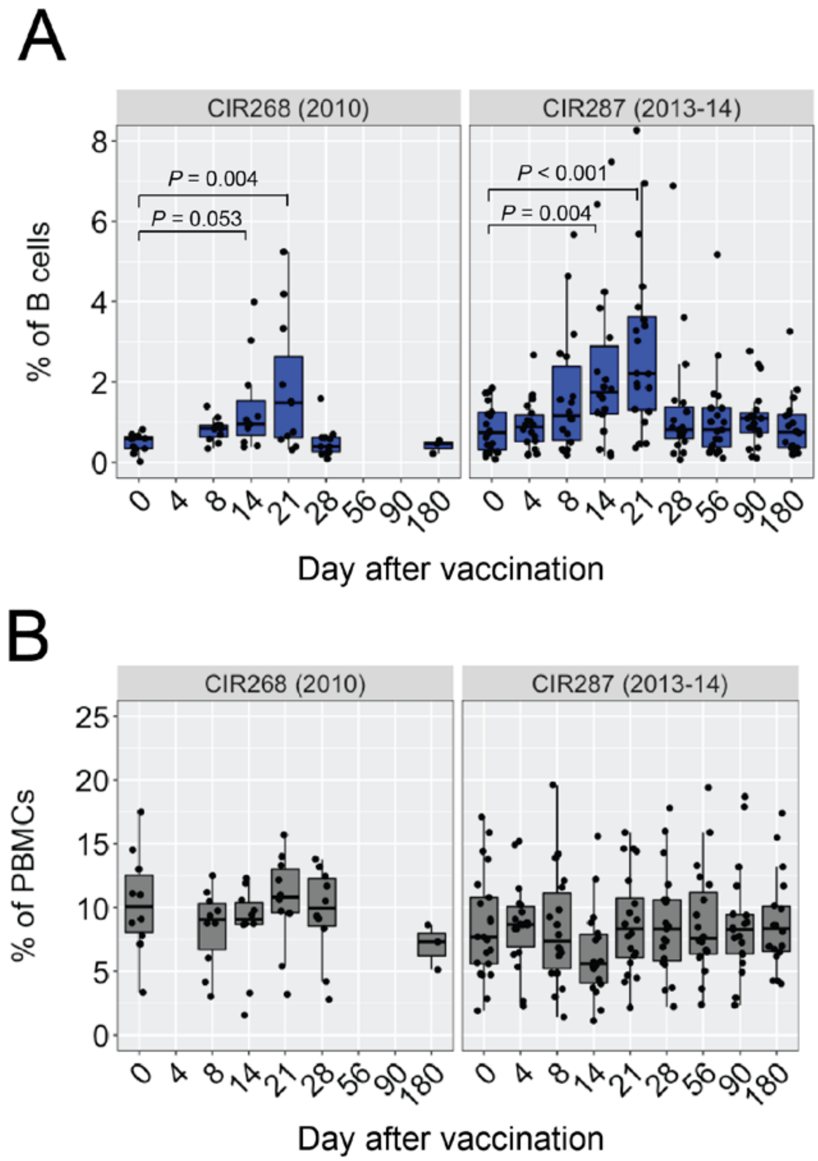


**Supplemental Figure 2 | Plasmablast and B cell frequencies after TV003 vaccination across two different clinical trials.** The TV003 vaccine trials CIR268 and CIR287 were conducted in 2010 and in 2013-14, respectively. (**A**) Plasmablast and (**B**) total B cell frequencies from each trial are summarized in boxplots with 25-75$^{th}$ percentiles as boxes, whiskers are 5-95$^{th}$ percentiles, and line is median. The plasmablast data were compared across the trials by linear means ANOVA with Tukey post-test, CIR268, n = 10 subjects per timepoint; CIR287, n = 21 subjects per timepoint). Related to Figure 1.

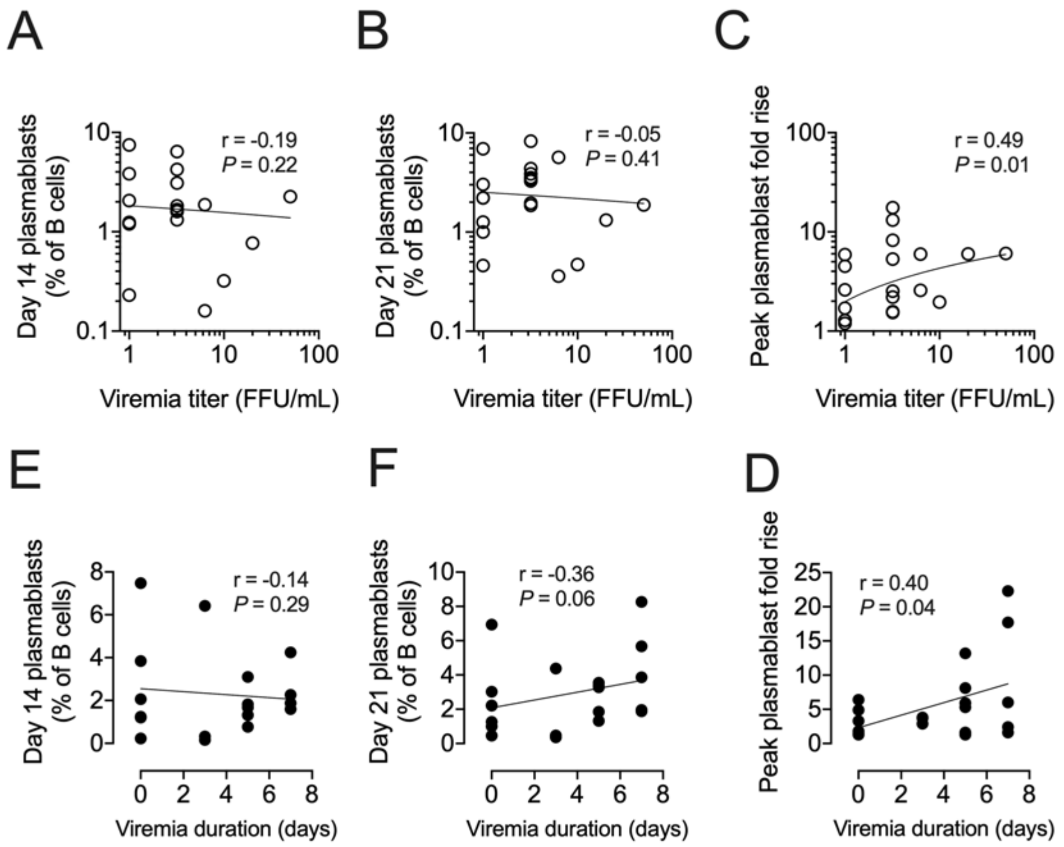


**Supplemental Figure 3 | Peak plasmablast induction and TV003 vaccine viral replication.** The relationship between peak vaccine virus titer and (**A**) Day 14 or (**B**) Day 21 plasmablast frequencies or (**C**) peak plasmablasts (Days 0-28) after TV003 vaccination are shown. Correlation analyses of vaccine viremia duration with (**D**) peak plasmablast fold-rise, (**E**) Day 14 and (**F**) Day 21 plasmablast frequencies. Spearman correlation coefficients and *P*-values are reported. Related to Figure 2.

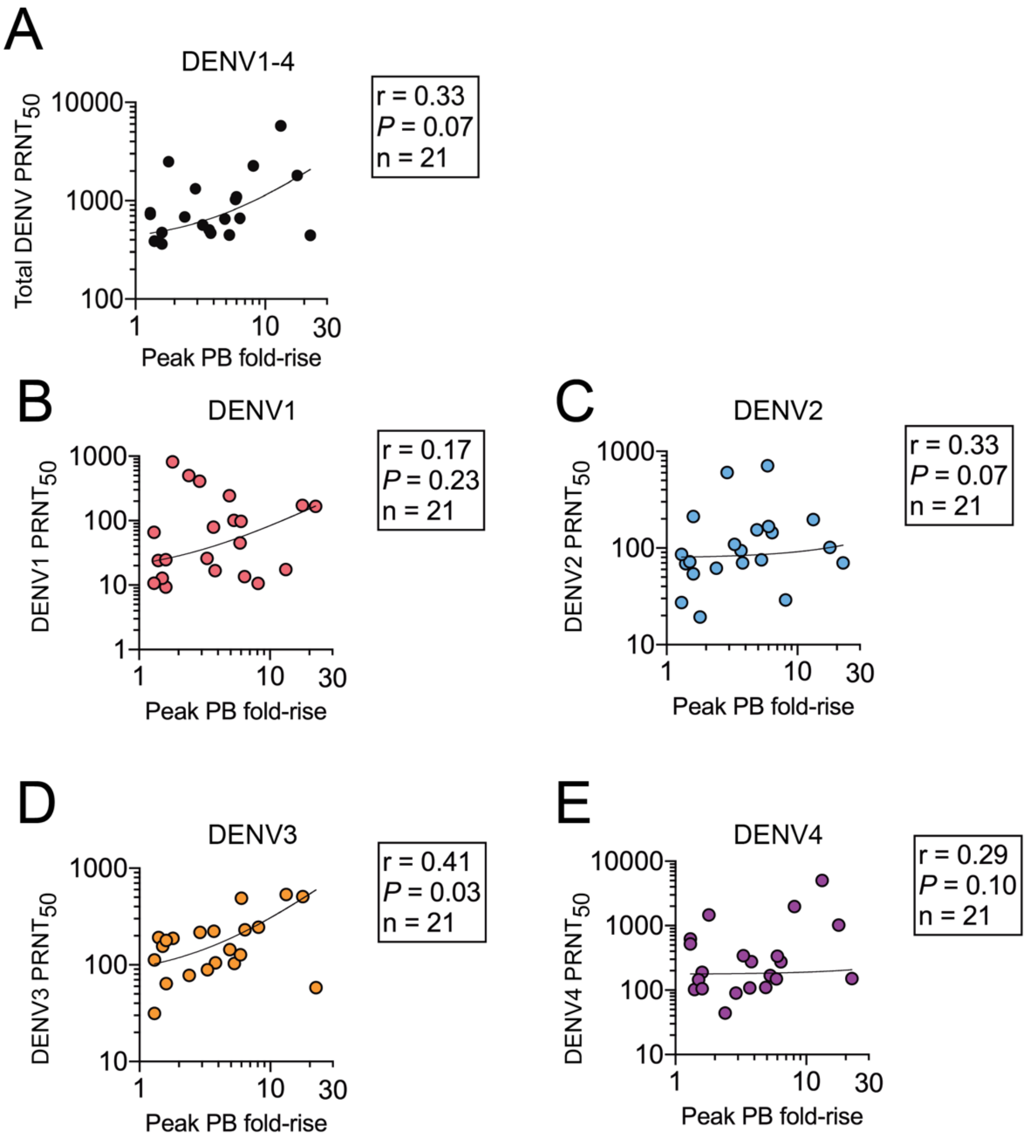


**Supplemental Figure 4 | Correlation of peak plasmablast induction by TV003 and the DENV neutralizing antibody response.** (**A-F**) The peak plasmablast response (Days 0-28) (expressed as fold-rise at peak compared to baseline) is plotted against the (**A**) sum of peak DEN1-4 $PRNT_{50}$ or against individual (**B**) DEN1, (**C**) DEN2, (**D**) DEN3, or (**E**) DEN4 peak $PRNT_{50}$ values induced within 180 days after TV003. Spearman correlation coefficients and *P*-values are reported. CIR287 trial data. Related to Figure 2.

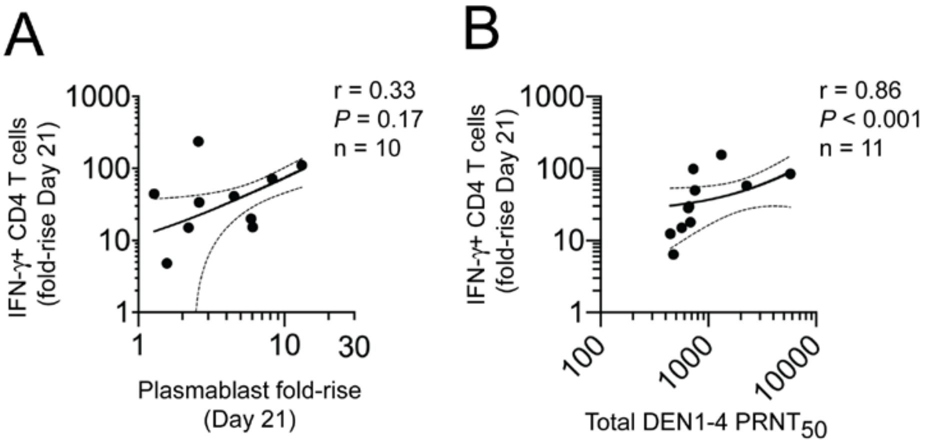


**Supplemental Figure 5 | Relationships between TV003-induced IFN-γ+ CD4+ T cells, plasmablasts, and serum neutralizing antibodies after TV003 vaccination. (A)** Total plasmablasts and DENV-specific IFN-γ+ CD4+ cells were measured ex vivo in subjects vaccinated with TV003. The fold rise in plasmablasts at day 21 after vaccination versus baseline is plotted against the day 21 fold-rise in DENV-specific IFN-γ+ CD4+ T cells. **(B)** The DENV-specific overall peak DENV serum neutralizing antibody titers (day 28 – 180 post-vaccination) are plotted against day 21 IFN-γ+ CD4+ T cell fold increase. Spearman correlation coefficients and *P*-values are reported. 95% confidence intervals shown in dashed lines. CIR287 trial data. Related to Figures 1,2.

**Supplemental Table S1. Peak plasmablast responses to TV003.**
Related to Figure 1.

| | | | Plasmablast fold-rise from Day 0 through Day 28 | |
|---|---|---|---|---|
| | **Subject** | **Peak frequency** | **Day 21** | **Peak (day)** |
| CIR268 (2010) | 268-A | 0.8% | 3.5× | 4.5× (8) |
| | 268-B | 3.0% | ≈ | 6.0× (14) |
| | 268-C | 1.5% | 3.0× | 3.0× (21) |
| | 268-D | 1.5% | 4.4× | 4.4× (21) |
| | 268-E | 4.2% | 8.6× | 8.6× (21) |
| | 268-F | 0.75% | 2.3× | 2.3× (21) |
| | 268-G | 4.2% | 6.0× | 6.0× (21) |
| | 268-H | 1.4% | ≈ | 2.0× (8) |
| | 268-I | 2.0% | 3.0× | 3.0× (21) |
| | 268-J | 1.0% | 17.0× | 47.0× (14) |
| CIR287 (2013-14) | 287-A | 7% | 6.1× | 22.3× (28) |
| | 287-B | 7% | 2.5× | 3.7× (14) |
| | 287-C | 0.5% | 5.9× | 6.4× (4) |
| | 287-D | 0.9% | 2.0× | 3.8× (28) |
| | 287-E | 4% | 2.6× | 3.3× (14) |
| | 287-F | 3.5% | 5.3× | 5.3× (21) |
| | 287-G | 2.0% | 1.6× | 1.6× (21) |
| | 287-H | 1.8% | 6.0× | 6.0× (21) |
| | 287-I | 5.5% | 4.5× | 4.9× (14) |
| | 287-J | 3.5% | 8.2× | 8.2× (21) |
| | 287-K | 1.3% | 1.8× | 1.8× (21) |
| | 287-L | 2.6% | 1.2× | 1.4× (8) |
| | 287-M | 1.2% | 1.3× | 1.5× (14) |
| | 287-N | 3.3% | 13.2× | 13.2× (21) |
| | 287-O | 1.9% | 1.6× | 1.6× (21) |
| | 287-P | 5.7% | 6.0× | 6.0× (21) |
| | 287-Q | 4.2% | 2.2× | 2.4× (14) |
| | 287-R | 2.2% | 1.3× | 1.3× (21) |
| | 287-S | 8.3% | 17.6× | 17.6× (21) |
| | 287-T | 0.4% | 2.6× | 2.6× (21) |
| Summary | | | # of subjects | |
| | Day 4 peak | | 1 | |
| | Day 8 peak | | 3 | |
| | **Day 14 peak** | | **7** | |
| | **Day 21 peak** | | **17** | |
| | Day 28 peak | | 2 | |

**Supplemental Table S2. Memory B cell 6xL immortalization efficiency**
Related to Figure 4.

| Subject ID | # MBC transduced | Transduction efficiency % 6XL-GFP+ (of CD19+) | Immortalized cells sorted | # DENV2-positive cultures | % DENV-specific MBC (of 6XL+ MBC) |
|---|---|---|---|---|---|
| **287-A** | 5,775 | 67% | 9,000 | 36 | 0.40% |
| **287-D** | 9,806 | 65% | 9,000 | 6 | 0.07% |
| **287-J** | 9,359 | 94% | 6,000 | 3 | 0.05% |
| **287-K** | 20,461 | 93% | 6,000 | 13 | 0.22% |
| **287-L** | 13,084 | 54% | 6,000 | 16 | 0.27% |
| **287-M** | 6,236 | 58% | 6,000 | 2 | 0.03% |
| **287-N** | 9,004 | 26% | 6,000 | 6 | 0.10% |
| **287-O** | 9,419 | 756% | 6,000 | 19 | 0.32% |
| **287-Q** | 3,429 | 96% | 6,000 | 2 | 0.03% |
| **287-S** | 5,576 | 67% | 9,000 | 10 | 0.11% |
| **287-T** | 17,916 | 42% | 6,000 | 4 | 0.07% |
| **Average** | 10,006 | 67% | 6,818 | 11 | 0.15% |

**Table S3. Correlation matrix of post-TV003 DENV2-specific MBC frequencies with plasmablast and serum neutralizing antibodies.** Related to Figure 4.

| Freq. DENV2+ MBCs vs: | Plasmablasts | | | | | | Serum neutralizing antibodies | | | | |
|---|---|---|---|---|---|---|---|---|---|---|---|
| | Frequency (% of B cells) | | | Fold-rise versus baseline | | | Peak post- TV003 (days 0-180) | | | | |
| | Day 14 | Day 21 | Peak | Day 14 | Day 21 | Peak | DEN1 | DEN2 | DEN3 | DEN4 | Total |
| Spearman R | 0.32 | 0.22 | 0.22 | –0.11 | –0.12 | –0.10 | 0.26 | –0.03 | 0.32 | 0.01 | -0.12 |
| *P*-value | 0.37 | 0.51 | 0.51 | 0.76 | 0.72 | 0.78 | 0.43 | 0.94 | 0.33 | 0.98 | 0.72 |
| n | 10 | 11 | 11 | 10 | 11 | 11 | 11 | 11 | 11 | 11 | 11 |
